# miR-302a/b/d-3p Differentially Expressed During Frontonasal Development Is Sensitive to Retinoic Acid Exposure

**DOI:** 10.3390/cells14141068

**Published:** 2025-07-11

**Authors:** Chihiro Iwaya, Akiko Suzuki, Goo Jun, Junichi Iwata

**Affiliations:** 1 Department of Orthodontics and Pediatric Dentistry, School of Dentistry, University of Michigan, Ann Arbor, MI 48109, USA; ciwaya@umich.edu (C.I.); akikosuz@umich.edu (A.S.); 2 Department of Epidemiology, School of Public Health, The University of Texas Health Science Center at Houston, Houston, TX 77030, USA; goo.jun@uth.tmc.edu; 3 Department of Diagnostic & Biomedical Sciences, School of Dentistry, The University of Texas Health Science Center at Houston, Houston, TX 77054, USA

**Keywords:** craniofacial development, microRNA, retinoic acid

## Abstract

Any failure in frontonasal development can lead to malformations at the middle facial region, such as frontonasal dysplasia, midfacial clefts, and hyper/hypotelorism. Various environmental factors influence morphogenesis through epigenetic regulations, including the action of noncoding microRNAs (miRNAs). However, it remains unclear how miRNAs are involved in the frontonasal development. In our analysis of publicly available miRNA-seq and RNA-seq datasets, we found that miR-28a-5p, miR-302a-3p, miR-302b-3p, and miR-302d-3p were differentially expressed in the frontonasal process during embryonic days 10.5 to 13.5 (E10.5–E13.5) in mice. Overexpression of these miRNAs led to a suppression of cell proliferation in cultured mouse embryonic frontonasal mesenchymal (MEFM) cells as well as in O9-1 cells, a cranial neural crest cell line. Through advanced bioinformatic analyses and miRNA-gene regulation assays, we identified that miR-28a-5p regulated a total of 25 genes, miR-302a-3p regulated 23 genes, miR-302b-3p regulated 22 genes, and miR-302d-3p regulated 20 genes. Notably, the expression of miR-302a/b/d-3p—unlike miR-28a-5p—was significantly upregulated by excessive exposure to *all-trans* retinoic acid (*at*RA) that induces craniofacial malformations. Inhibition of these miRNAs restored the reduced cell proliferation caused by *at*RA by normalizing the expression of target genes associated with frontonasal anomalies. Therefore, our findings suggest that miR-302a/b/d-3p plays a crucial role in the development of frontonasal malformations.

## 1. Introduction

Midfacial development begins with the induction of the frontonasal process and two bilateral nasal placodes at embryonic day E10.0 in mice and during the 5th week of gestation in humans. From the frontonasal process, the lateral and medial nasal processes emerge, forming nasal pits at the center. By E11.0, these structures start to fuse, creating one continuous formation that merges with the maxillary processes along the midline by E13.5 and form the upper lip in mice. Any disruption in frontonasal development, including cell migration, patterning, and cell proliferation, can lead to malformations such as midfacial clefts, hypoplasia, and hypertelorism [1].

Several environmental factors have been associated with the development of frontonasal malformations [2,3]. For instance, cigarette smoking, maternal alcohol consumption, and poor diets—specifically those lacking in zinc, myoinositol, folic acid, and retinoic acid (RA), a derivative of vitamin A—are known risk factors [3]. High doses of ethanol during pregnancy have been shown to cause anterior neural plate defects and cleft lips due to increased cell death in mice [4,5]. Various signaling pathways, including the sonic hedgehog (SHH) and RA pathways, become dysregulated under these conditions. For example, SHH signaling is disrupted in cases of fetal alcohol syndrome [5,6]. Both excessive and insufficient RA levels—originating from the ventral epithelium of the frontonasal process and affecting cranial neural crest (CNC) cells [7]—can lead to craniofacial developmental defects [2,8,9]. Severe vitamin A deficiency, often due to maternal malnutrition, raises the risk of frontonasal developmental anomalies [10]. Conversely, excess vitamin A intake during pregnancy called to hypervitaminosis A, particularly through the medication isotretinoin (an RA antagonist) and vitamin A supplements, can result in a condition known as retinoid syndrome in infants, which is characterized by growth retardation, hydrocephaly, microcephaly, intellectual disabilities, cardiovascular defects, and craniofacial dysmorphisms, including cleft lip, cleft palate, hypertelorism, and midfacial hypoplasia [10,11,12].

While numerous environmental factors have been shown to contribute to frontonasal malformations, the specific mechanisms by which they operate remain unclear. Epigenetic factors, such as microRNAs (miRNAs)—which are short noncoding RNAs that regulate gene expression at the post-transcriptional level—are influenced by environmental conditions, especially those that are pathogenic [13,14]. The accumulating evidence suggests that miRNAs play significant roles in craniofacial development [15,16,17,18]. However, which miRNAs are sensitive to environmental changes and how miRNA–gene regulatory networks contribute to the phenotype remain elusive in frontonasal developmental anomalies. This study aims to identify miRNAs associated with frontonasal development and to investigate how excessive *all-trans* retinoic acid (*at*RA) disturbs frontonasal development.

## 2. Materials and Methods

### 2.1. Bioinformatic Analysis

miRNA-seq and RNA-seq datasets from the developing frontonasal processes of C57BL/6J mouse embryos were analyzed. The datasets include E10.5 miRNA-seq (FB00000662.01 and FB00000492.01), E10.5 RNA-seq (FB00000347 and FB00000867), E11.5 miRNA-seq (FB00000663.01 and FB00000492.01), E11.5 RNA-seq (FB00000867), E12.5 miRNA-seq (FB00000493.01 and FB00000664.01), E12.5 RNA-seq (FB00000867), E13.5 miRNA-seq (FB00000346 and FB00000665.01), and E13.5 RNA-seq (FB00000278). These datasets are available in the FaceBase database. All miRNA FASTQ files were re-mapped using sRNAtoolbox [19]. Replica samples with fewer reads than the minimum threshold were excluded from the analyses. All total RNA-seq mRNA data were generated by mapping reads in FASTQ files to the GRCm38 reference sequence using STAR aligner with “--runMode alignReads --outSAMtype BAM SortedByCoordinate --quantMode TranscriptomeSAM GeneCounts” options [20] and gene expression levels were calculated by RSEM with the “rsem-calculate-expression” option on the BAM file generated by STAR [21]. Differential expression analyses were conducted using the edgeR and Linear Models for Microarray (LIMMA) package [22]. FDR-adjusted *p* value < 0.05 using Benjamini–Hochberg procedure was considered statistically significant and used for the threshold.

### 2.2. Cell Culture

The animal protocol (PRO00011979) was approved by the Animal Welfare Committee (AWC) and the Institutional Animal Care and Use Committee (IACUC) of the University of Michigan. All mice were bred under pathogen-free conditions, with free access to clean water and food, and maintained on a 12 h light/12 h dark cycle. Carbon dioxide (CO_2_) inhalation was used for euthanasia. Primary mouse embryonic frontonasal mesenchymal (MEFM) cells were isolated from the frontonasal process of E10.5 C57BL/6J mice. Tissues from three embryos were pooled and treated as a single sample. The frontonasal process was dissected in sterile Dulbecco’s Phosphate-Buffered Saline (D-PBS) and then suspended into single-cell suspensions using 0.25% trypsin and 0.05% EDTA for 10 min at 37 °C in an atmosphere of 5% CO_2_. MEFM cells were maintained in Dulbecco’s Modified Eagle’s Medium (DMEM) containing high glucose (Sigma-Aldrich Inc., St. Louis, MO, USA) supplemented with 10% fetal bovine serum, penicillin and streptomycin, β-mercaptoethanol, MEM nonessential amino acids, L-glutamine, and sodium pyruvate at 37 °C in a humidified atmosphere with 5% CO_2_. The O9-1 cells (SCC049, MilliporeSigma, Burlington, MA, USA) were cultured in the complete embryonic stem (ES) cell medium (ES-101-B, Sigma-Aldrich) at 37 °C in a humidified atmosphere with 5% CO_2_.

### 2.3. Cell Proliferation and Cell Death Assay

MEFM or O9-1 cells were treated with a mimic for negative control (4464061, Thermo Fisher Scientific, Waltham, MA, USA), miR-28a-5p, miR-302a-3p, miR-302b-3p, or miR-302d-3p (4464066, ThermoFisher Scientific) or an inhibitor for negative control (4464079, Thermo Fisher Scientific), miR-28a-5p, miR-302a-3p, miR-302b-3p, or miR-302d-3p (4464084, Thermo Fisher Scientific). Cell proliferation was measured using the Cell Counting Kit 8 (Dojindo Molecular Technologies, Inc., Rockville, MD, USA) at 24, 48, or 72 h after each treatment (n = 6 per group). For the *at*RA exposure experiments, MEFM or O9-1 cells were treated with 0 to 50 μM *at*RA for 3 days (n = 6 per group). Cell death was analyzed with TUNEL assays (Click-iT^TM^ Plus TUNEL assay kit; C10619, Thermo Fisher Scientific), following the manufacturer’s protocol (n = 6 per group).

### 2.4. Cell Migration Assay

MEFM or O9-1 cells were seeded in 12-well plates at a concentration of 1 × 10^5^ cells/well for MEFM cells and 5 × 10^4^ cells/well for O9-1 cells in 2 mL of the appropriate growth medium. Once the cells reached 70% confluence, they were treated with either a negative control mimic or one of the miR-28a-5p, miR-302a-3p, miR-302b-3p, or miR-302d-3p mimics. After 24 h of treatments, a straight-line scratch was created by scraping the cell layer using a P100 pipette tip. Cell migration was then monitored 24 and 48 h after scratching using a phase-contrast microscope (CKY53, Olympus, Center Valley, PA, USA), with triplicate samples for each treatment (n = 3).

### 2.5. Quantitative RT-PCR

Total RNA was extracted from the frontonasal process of C57BL/6J embryos at embryonic days E10.5 to E13.5, as well as from MEFM and O9-1 cells after 24 h of treatment with either a mimic or an inhibitor for miR-28a-5p, miR-302a-3p, miR-302b-3p, or miR-302d-3p, or negative control. The gene expression was analyzed using quantitative RT-PCR (qRT-PCR) (n = 6). The PCR primers utilized in this study are listed in Table S1. Each gene’s expression was normalized to *Gapdh* expression. The miRNA expression was measured using TaqMan Fast Advanced Master Mix and TaqMan Advanced miRNA cDNA Synthesis Kit (Thermo Fisher Scientific), following the manufacturer’s protocol (n = 6). Probes for miR-92b-3p (mmu481277_mir), miR-26a-5p (477995_mir), miR-28a-5p (mmu482619_mir), miR-302a-3p (mmu482946_mir), miR-302b-3p (mmu481677_mir), and miR-302d-3p (mmu478237_mir) were purchased from Thermo Fisher Scientific.

### 2.6. Statistical Analysis

All experimental results were obtained from at least three independent experiments and analyzed using Prism software (GraphPad Software, Boston, MA, USA, version 10.5.0). A *p*-value of less than 0.05 was considered statistically significant. A two-tailed non-parametric Student’s *t*-test was used for comparisons between two groups, while one-way analysis of variance (ANOVA) with the Tukey–Kramer post hoc test was employed for multiple comparisons. For cell proliferation assays, a two-way ANOVA was conducted. The data are presented as mean ± standard deviation (SD).

## 3. Results

### 3.1. miRNAs Expressed in the Frontonasal Process in a Temporal-Specific Manner During Frontonasal Development

**Figure 1:**
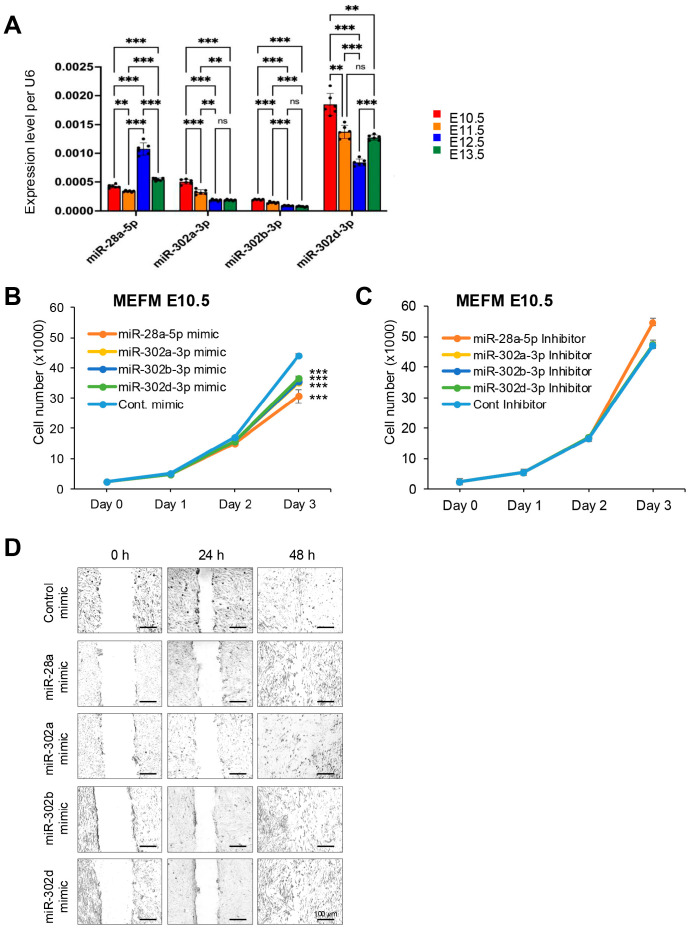
Expression level and function of miR-28-5p, miR-302a-3p, miR-302d-3p, and miR-302d-3p on cell proliferation. (**A**) Quantitative RT-PCR for expression of miR-28-5p, miR-302a-3p, miR-302d-3p, and miR-302d-3p in the frontonasal process at E10.5, E11.5, E12.5, and E13.5. ** *p* < 0.01, *** *p* < 0.001. ns: not significant. (**B**,**C**) Cell proliferation assays in MEFM cells treated with the indicated miRNA mimic (**B**) or inhibitor (**C**). *** *p* < 0.001. Each treatment group was compared to control. (**D**) Cell migration assays at 0, 24, and 48 h in MEFM cells treated with the indicated miRNA mimic. Scale bars, 100 µm.

To identify differentially expressed miRNAs in the frontonasal process during development, we analyzed RNA-seq datasets available in the FaceBase database (https://www.facebase.org, accessed on 15 November 2017). We observed some interesting miRNA expression patterns across different developmental stages. For instance, miR-28a-5p was upregulated at E12.5 and then downregulated at E13.5. Expression of miR-302a/b/d-3p was gradually downregulated over the time E10.5 to E13.5 (Table 1). Developmental stage E10.5-E11.5 is critical for extensive outgrowth and fusion with other primordia in frontonasal development [1]. miR-28a-5p and miR-302a/b/d-3p were significantly downregulated at E11.5 compared to E10.5 (Table 1), suggesting that target genes of these miRNAs can upregulate at E11.5 compared to E10.5. To confirm the expression level of these miRNAs during mouse frontonasal development, we conducted qRT-PCR analyses for each miRNA using the developing frontonasal prominence of C57BL/6J mice at E10.5, E11.5, E12.5, and E13.5 (Figure 1A). Next, to identify genes targeted by these miRNAs in frontonasal development, we analyzed the mRNA expression data available at Gene Expression Omnibus (GEO, accession number: GSE62214). First, to identify mRNAs anti-correlated with the miRNA expression, we compared differentially expressed genes and miRNAs using the GEO2R web application and identified 3636 probes differentially expressed between E10.5 and E11.5 and 2259 probes differentially expressed between E11.5 and E12.5. Among them, 127 miRNAs were upregulated between E10.5 and E11.5 but downregulated between E11.5 and E12.5. Although 257 genes showed a prediction score of 50 or greater for miR-28a-5p in the miRDB database (https://mirdb.org, accessed on 14 December 2017), none of them overlapped with the 127 anti-correlated miRNAs. We therefore analyzed the datasets separately at E10.5/E11.5 and E11.5/E12.5 for miR-28a-5p and found that 24 and 14 genes verified with both the prediction and anti-correlated expression at E10.5/E11.5 and E11.5/E12.5, respectively (Table 2). Similarly, expression of 31 genes was anti-correlated in miR-302a/b/d3p. Note that 30 out of 31 genes, except *Adamts5* only in miR-302d-3p, were overlapped between miR-302a-3p, miR-302b-3p, and miR-302d-3p (Table 2).

### 3.2. Overexpression of miR-28a-5p, miR-302a-3p, miR-302b-3p, and miR-302d-3p Inhibits Cell Proliferation Through Downregulation of Genes Related to Frontonasal Malformations in MEFM and O9-1 Cells

We investigated the functional role of specific miRNAs in cell proliferation and migration by using a specific mimic for each miRNA in MEFM and O9-1 cells. Our results revealed that the overexpression of these miRNAs significantly inhibited cell proliferation in both MEFM and O9-1 cells (Figure 1B and Figure S1A). In contrast, the application of inhibitors for these miRNAs did not have any effect on cell proliferation (Figure 1C and S1B). In addition, these mimic treatments did not influence cell migration in vitro, as assessed by scratch assays (Figure 1D and Figure S1C). Therefore, we conclude that the overexpression of miR-28a-5p, miR-302a-3p, miR-302b-3p, and miR-302d-3p can inhibit cell proliferation in cultured MEFM and O9-1 cells.

**Figure 2:**
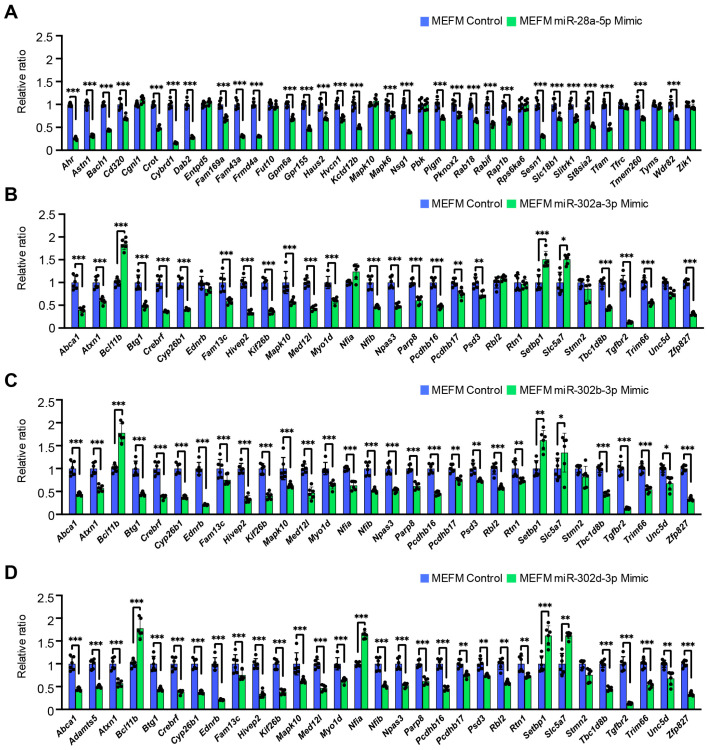
Effect of each miRNA mimic on predicted target gene expression in MEFM cells. (**A**–**D**) Quantitative RT-PCR for target genes in MEFM cells treated with miR-28a-5p mimic (**A**), miR-302a-3p mimic (**B**), miR-302b-3p mimic (**C**), and miR-302d-3p mimic (**D**) for 24 h. * *p* < 0.05, ** *p* < 0.01, *** *p* < 0.001. Each treatment group was compared with negative control.

Next, we validated the predicted miRNA–gene regulation for each miRNA using qRT-PCR for the genes in MEFM and O9-1 cells treated with specific mimic for each miRNA (Figure 2 and Figure S2). We found that miR-28a-5p mimic significantly downregulated the expression of *Ahr*, *Astn1*, *Bach1*, *Cd320*, *Crot*, *Cybrd1*, *Dab2*, *Fam169a*, *Fam43a*, *Frmd4a*, *Gpm6a*, *Gpr155*, *Kctd12b*, *Nsg1*, *Pigm*, *Pknox2*, *Rab18*, *Rabif*, *Rap1b*, *Sesn1*, *Slc18b1*, *Slitrk1*, *St8sia2*, *Tmem260*, and *Wdr82* in both MEFM and O9-1 cells (Figure 2A and Figure S2A). A miR-302a-3p mimic significantly downregulated expression of *Abca1*, *Atxn1*, *Btg1*, *Crebrf*, *Cyp26b1*, *Fam13c*, *Hivep2*, *Kif26b*, *Mapk10*, *Med12l*, *Myo1d*, *Nfib*, *Npas3*, *Parp8*, *Pcdhb16*, *Psd3*, *Setbp1*, *Slc5a7*, *Tbc1d8b*, *Tgfbr2*, *Trim66*, and *Zfp827* in both MEFM and O9-1 cells (Figure 2B and Figure S2B). A miR-302b-3p mimic significantly downregulated expression of *Abca1*, *Atxn1*, *Btg1*, *Crebrf*, *Cyp26b1*, *Ednrb*, *Fam13c*, *Hivep2*, *Kif26b*, *Mapk10*, *Med12l*, *Myo1d*, *Nfia*, *Nfib*, *Npas3*, *Parp8*, *Pcdhb16*, *Pcdhb17*, *Psd3*, *Rbl2*, *Setbp1*, *Slc5a7*, *Tbc1d8b*, *Tgfbr2*, *Trim66*, *Unc5d*, and *Zfp827* in both MEFM and O9-1 cells (Figure 2C and Figure S2C). A miR-302d-3p mimic downregulated expression of *Abca1*, *Adamts5*, *Atxn1*, *Btg1*, *Crebrf*, *Cyp26b1*, *Ednrb*, *Fam13c*, *Hivep2*, *Kif26b*, *Mapk10*, *Med12l*, *Myo1d*, *Nfia*, *Nfib*, *Npas3*, *Parp8*, *Pcdhb16*, *Pcdhb17*, *Psd3*, *Rbl2*, *Rtn1*, *Setbp1*, *Slc5a7*, *Tbc1d8b*, *Tgfbr2*, *Trim66*, *Unc5d*, and *Zfp827* in both MEFM and O9-1 cells (Figure 2D and Figure S2D).

**Figure 3:**
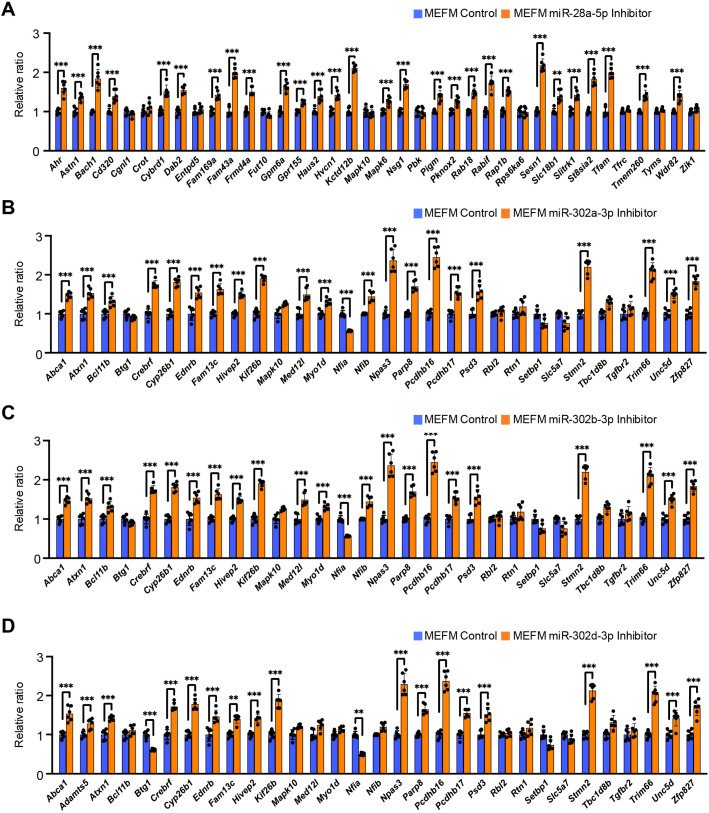
Effect of each miRNA inhibitor on predicted target gene expression in MEFM cells. (**A**–**D**) Quantitative RT-PCR for target genes in MEFM cells treated with miR-28a-5p inhibitor (**A**), miR-302a-3p inhibitor (**B**), miR-302b-3p inhibitor (**C**), and miR-302d-3p inhibitor (**D**) for 24 h. ** *p* < 0.01, *** *p* < 0.001. Each treatment group was compared with negative control.

To test a dose-dependent miRNA effect on gene expression, we treated the cells with each miRNA inhibitor and found that miR-28a-5p inhibitor significantly upregulated expression of *Ahr*, *Astn1*, *Bach1*, *Cd320*, *Cybrd1*, *Dab2*, *Fam169a*, *Fam43a*, *Fmd4a*, *Gpm6a*, *Gpr155*, *Haus2*, *Hvcn1*, *Kctd12b*, *Mapk6*, *Nsg1*, *Pigm*, *Pknox2*, *Rab18*, *Rabif*, *Rap1b*, *Sesn1*, *Slc18b1*, *Slitrk1*, *St8sia2*, *Tfam, Trem260*, and *Wdr82* in both MEFM and O9-1 cells (Figure 3A and Figure S3A). A miR-302a-3p inhibitor significantly upregulated expression of *Abca1*, *Atxn1*, *Bcl11b*, *Crebrf*, *Cyp26b1*, *Ednrb*, *Fam13c*, *Hivep2*, *Kif26b*, *Med12l*, *Myo1d*, *Nfia*, *Nfib*, *Npas3*, *Parp8*, *Pcdhb16*, *Pcdhb17*, *Psd3*, *Stmn2*, *Trim66*, *Unc5d*, and *Zfp827* in both MEFM and O9-1 cells (Figure 3B and Figure S3B). A miR-302b-3p inhibitor significantly upregulated expression of *Abca1*, *Atxn1*, *Bcl11b*, *Crebrf*, *Cyp26b1*, *Ednrb*, *Fam13c*, *Hivep2*, *Kif26b*, *Med12l*, *Myo1d*, *Nfia*, *Nfib*, *Npas3*, *Parp8*, *Pcdhb16*, *Pcdhb17*, *Psd3*, *Stmn2*, *Trim66*, *Unc5d*, and *Zfp827* in both MEFM and O9-1 cells (Figure 3C and Figure S3C). A miR-302d-3p inhibitor significantly upregulated expression of *Abca1*, *Adamts5*, *Atxn1*, *Btg1*, *Crebrf*, *Cyp26b1*, *Ednrb*, *Fam13c*, *Hivep2*, *Kif26b*, *Nfia*, *Npas3*, *Parp8*, *Pcdhb16*, *Pcdhb17*, *Psd3*, *Stmn2*, *Trim66*, *Unc5d*, and *Zfp827* in both MEFM and O9-1 cells (Figure 3D and Figure S3D). Taken together, the expression of 20 genes (*Ahr*, *Astn1*, *Bach1*, *Cd320*, *Crot*, *Cybrd1*, *Dab2*, *Fam43a*, *Fmd4a*, *Gpm6a*, *Kctd12b*, *Nsg1*, *Rab18*, *Rabif*, *Rap1b*, *Sesn1*, *Slc18b1*, *Slitrk1*, *St8sia2*, *Trem260*, and *Wdr82*) were regulated by miR-28a-5p, 21 genes (*Abca1*, *Atxn1*, *Crebrf*, *Cyp26b1*, *Ednrb*, *Fam13c*, *Hivep2*, *Kif26b*, *Med12l*, *Myo1d*, *Nfia*, *Nfib*, *Npas3*, *Parp8*, *Pcdhb16*, *Pcdhb17*, *Psd3*, *Stmn2*, *Trim66*, *Unc5d*, and *Zfp827*) by miR-302a-3p, 21 genes (*Abca1*, *Atxn1*, *Crebrf*, *Cyp26b1*, *Ednrb*, *Fam13c*, *Hivep2*, *Kif26b*, *Med12l*, *Myo1d*, *Nfia*, *Nfib*, *Npas3*, *Parp8*, *Pcdhb16*, *Pcdhb17*, *Psd3*, *Stmn2*, *Trim66*, *Unc5d*, and *Zfp827*) by miR-302b-3p, and 20 genes (*Abca1*, *Adamts5*, *Atxn1*, *Btg1*, *Crebrf*, *Cyp26b1*, *Ednrb*, *Fam13c*, *Hivep2*, *Kif26b*, *Nfia*, *Npas3*, *Parp8*, *Pcdhb16*, *Pcdhb17*, *Psd3*, *Stmn2*, *Trim66*, *Unc5d*, and *Zfp827*) by miR-302d-3p in both MEFM and O9-1 cells in a dose-dependent manner.

### 3.3. Identification of miRNAs Altered by Excessive atRA

**Figure 4:**
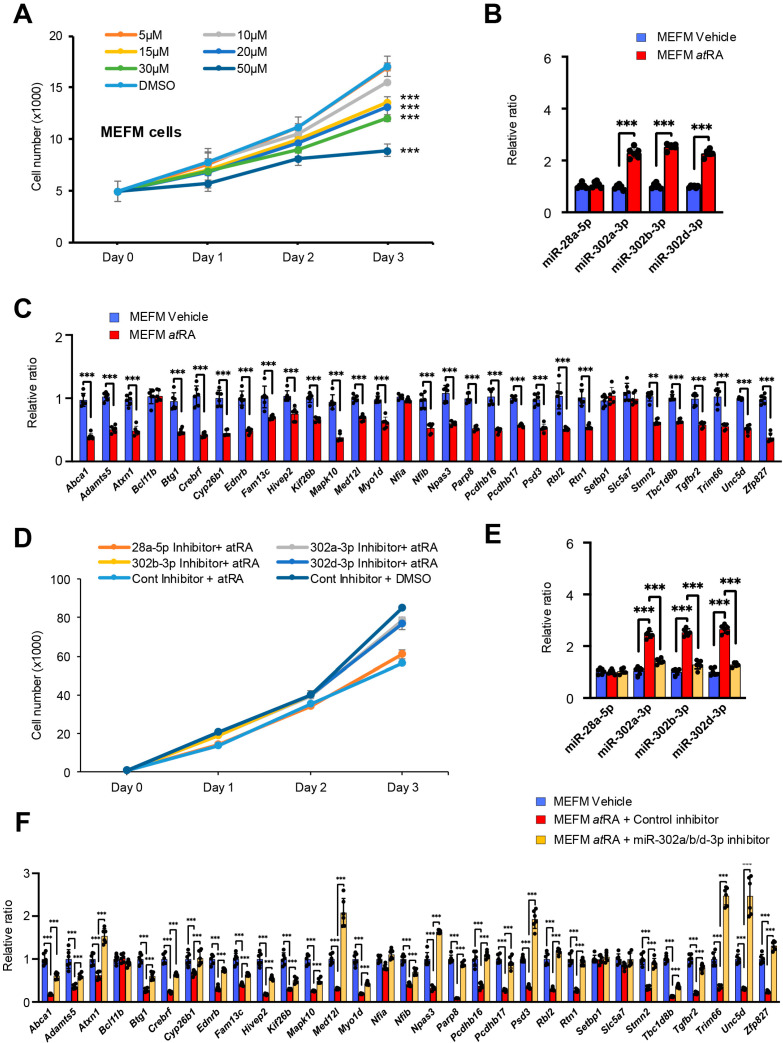
miR-302a/b/d-3p inhibitors restore *at*RA-induced cell proliferation inhibition. (**A**) Cell proliferation assays in MEFM cells treated with *at*RA from 5 to 50 μM for three days. (**B**) Expression of miR-302 family and miR-28-5p in MEFM cells treated with 50 μM *at*RA. (**C**) Quantitative RT-PCR for target genes in MEFM cells treated with 50 μM *at*RA. ** *p* < 0.01, *** *p* < 0.001. Each treatment group was compared to a vehicle control group. (**D**) Cell proliferation assays in MEFM cells treated with a miRNA inhibitor for each miR-28a-5p and miR-302a/b/d-3p for three days under 50 μM *at*RA. (**E**) miRNA expression in MEFM cells treated with each miRNA inhibitor under 50 μM *at*RA. (**F**) Quantitative RT-PCR for target genes in MEFM cells treated with miR-302a/b/d-3p inhibitor under 50 μM *at*RA. *** *p* < 0.001. Each treatment group was compared to a negative control group.

Recent studies indicate that the expression of miRNAs is influenced by various factors, including the use of substances, cigarettes, and alcohol, as well as body weight, sex, age, and various diseases [23,24,25,26]. It is well established that an excess of vitamin A and its metabolite *at*RA during pregnancy can lead to teratogenic effects on craniofacial development. To determine whether excessive *at*RA induces the identified miRNAs in O9-1 and MEFM cells, we performed cell proliferation assays, TaqMan assays for miRNA expression, and qRT-PCR for the target genes of the miRNAs (Figure 4).

We found that cell proliferation was inhibited by *at*RA in a dose-dependent manner in MEFM and O9-1 cells (Figure 4A and Figure S4A). Interestingly, expression of miR-302a/b/d-3p, but not miR-28a-5p, was upregulated in MEFM and O9-1 cells treated with *at*RA compared to vehicle controls (Figure 4B and Figure S4B). We further confirmed that expression of several genes targeted by miR-302a/b/d-3p was significantly downregulated with *at*RA treatment in both MEFM and O9-1 cells (Figure 4C and Figure S4C). Finally, to test functional significance of miR-302a/b/d-3p in cell proliferation inhibition under *at*RA exposure, we examined cell proliferation in MEFM and O9-1 cells treated with a miR-302a/b/d-3p inhibitor under *at*RA condition and found that miR-302a/b/d-3p inhibitor ameliorated cell proliferation defect (Figure 4D and Figure S4D), miR-302a/b/d-3p expression (Figure 4E and Figure S4E), and downregulated gene expression (Figure 4F and Figure S4F). The expression of the miR-302 family was found to be upregulated following treatment with *at*RA. However, the miR-302a/b/d-3p mimic did not successfully suppress some candidate target genes, including *Bcl11b*, *Nfia*, *Setbp1*, and *Slc5a7* (Figure 1B–D). This suggests that multiple miRNAs may regulate the expression of these genes under *at*RA conditions. Notably, among the candidate genes, mice lacking *Tgfbr2* in neural crest cells (*Wnt1-Cre;Tgfbr2* conditional knockout) exhibit shortened maxilla, absence of the frontal bone, and cleft palate [27]. Taken together with our findings, these results suggest that excessive *at*RA may inhibit cell proliferation through the miR-302a/b/d-3p-*Tgfbr2* pathway.

## 4. Discussion

Excessive maternal exposure to *at*RA causes craniofacial anomalies in mice, indicating that mesenchymal cells derived from CNC cells are more sensitive to *at*RA compared to other cell lineages. Previous studies show that *at*RA suppresses cell proliferation in palatal mesenchyme by upregulating miR-124-3p and miR-340-5p in mice [18,28] and miR-4680-3p in humans [29]. Notably, high doses of *at*RA lead to cleft palate in mice but do not affect upper lip formation, whereas excessive phenytoin causes cleft lip by inhibiting cell proliferation in the maxillary and nasal prominences [30,31]. These findings suggest that sensitivity of CNC-derived mesenchymal cells to *at*RA is influenced by the timing and dosage of exposure as well as the location of the migrating cells. In this study, we found that excessive *at*RA disrupts proper frontonasal development by altering the expression of the miR-302 family, which is an intronic and embryonic stem (ES)-cell-specific miRNA [32]. Under normal physiological conditions, miR-302a/b/d-3p is consistently expressed at very low levels from E10.5 to E12.5 in the developing frontonasal process, as demonstrated in miR-302::GFP mouse embryos [33].

A previous study of human palatal mesenchymal cells indicates that *at*RA suppresses cell proliferation and enhances extracellular matrix (ECM) degradation by increasing the MMP2/TIMP2 expression ratio through the TGF-β/SMAD signaling pathway [34]. In a mouse embryonic palate explant model, defects in cell proliferation caused by *at*RA were rescued by folic acid supplementation, which normalized the expression of TGFB3 and TGFBR2 [35]. Furthermore, *at*RA encourages ES cell differentiation by modifying the expression of both genes and miRNAs [36]. The loss-of-function of miR-302 in mice (*miR-302* null mice) results in failure to close the neural tube due to thickened neuroepithelium, characterized by increased cell proliferation, decreased apoptosis, and premature neurogenesis/differentiation of neuroepithelial cells [33]. A recent study shows that loss of miR-302 upregulates glycolysis through the upregulated expression of *Pfkp*, *Pfkfb3*, and *Hk1*, which leads to a short cell cycle and increased cell proliferation during neural tube closure [37]. Treatments with a miR-302 antagonist suppress cell proliferation in human stem cells in a dose-dependent manner and inhibit teratoma formation in mouse xenografts by upregulating *AKT1* and *OCT4* [38]. In addition, the *has-miR-302* cluster suppresses ectodermal cell differentiation in human pluripotent stem cells [39]. These findings indicate that the miR-302 family significantly influences cell growth and survival. Some neural crest (NC) cells retain pluripotency even after migrating to their final destinations [40]. miR-302 expression is notably high in E7.5 epiblasts and E8.5 CNC cells, with a dramatic decrease occurring by E9.5. Intriguingly, miR-302-null embryos between E8.0 and E9.5 exhibit an increase in CNC cells along with early expression of *Sox9*, a key gene involved in CNC specification [41], implying that miR-302 is vital for both CNC cell fate determination and proliferation. Thus, the suppression of cell proliferation due to excessive *at*RA in CNC-derived cells is likely mediated by the miR-302 family and *Tgfbr2* downregulation. POU5F1 (a.k.a. OCT4) and SOX2, which are well-known stem cell makers, are essential for maintaining multipotency in CNC-derived cells [42,43,44]. The miR-302 promotor contains binding sites for POU5F1 and SOX2, suggesting that these factors can positively regulate miR-302 expression [45]. Moreover, miR-302 negatively regulates *Ccnd1* expression, leading to an accelerated transition of the cell cycle from G1 to S phase in human ES cells [45]. Thus, the miR-302 family can switch cell fate from the stem cell stage to the stages of proliferation and differentiation.

miRNAs are crucial for gene expression essential for development under normal physiological conditions. We hypothesized that, under pathological conditions, such as excessive *at*RA exposure, abnormal miRNAs, which are either not expressed or expressed at basal levels under normal conditions, are induced, leading to the suppression of genes essential for development. While miRNA inhibitors for miR-28a-5p and miR-302a/b/d-3p did not significantly affect cell proliferation, notable changes were observed in the expression of their predicted target genes. This raises questions about the functional relevance of these targets in the context of proliferation; therefore, we examined cell migration. However, we did not observe any significant changes in migration in cells treated with miRNA inhibitors. These results suggest that cell proliferation might be sufficiently robust in MEFM and O9-1 cells under these conditions. Furthermore, although miRNA inhibitors could cause non-physiological suppression of both high- and low-affinity target genes, the addition of inhibitors may not functionally affect cell proliferation beyond endogenous miRNA levels. On the other hand, when pathogenic miRNAs were inhibited, both the target gene expression and cell proliferation were normalized.

## 5. Conclusions

Our results indicate that miR-302a/b/d-3p expression is specifically upregulated under excessive *at*RA conditions, suggesting that they are pathogenic miRNAs induced by *at*RA, which lead to frontonasal hypoplasia and/or midline clefts.

## Figures and Tables

**Table 1:** miRNAs differentially expressed in different stages (E10.5, E11.5, E12.5, and E13.5) with FDR-adjusted *p*-value < 0.05. A: E10.5 vs. E11.5, B: E11.5 vs. E12.5, and C: E12.5 vs. E13.5.

**A. E10.5 vs. E11.5**		
miRNAs	logFC	FDR-Adjusted *p*-Value
mmu-miR-28a-5p	−4.37	1.86 × 10^−4^
mmu-miR-302b-3p	−2.79	3.13 × 10^−2^
mmu-miR-302a-3p	−2.76	3.13 × 10^−2^
mmu-miR-302d-3p	−2.65	3.42 × 10^−2^
mmu-miR-199a-5p	1.44	3.13 × 10^−2^
mmu-miR-125b-5p	1.79	3.14 × 10^−3^
mmu-miR-143-3p	2.14	1.86 × 10^−4^
mmu-miR-143-5p	2.68	3.75 × 10^−2^
mmu-miR-24-3p	2.88	3.14 × 10^−3^
**B. E11.5 vs. E12.5**		
miRNA	logFC	FDR-adjusted *p*-value
mmu-miR-300-5p	−5.39	1.74 × 10^−2^
mmu-miR-302d-3p	−3.88	1.30 × 10^−3^
mmu-miR-302a-3p	−3.71	3.03 × 10^−3^
mmu-miR-302b-3p	−3.71	3.03 × 10^−3^
mmu-miR-3063-3p	−3.56	1.30 × 10^−3^
mmu-miR-341-3p	−2.37	6.18 × 10^−4^
mmu-miR-20b-5p	−1.99	4.59 × 10^−2^
mmu-miR-335-3p	−1.73	4.22 × 10^−3^
mmu-miR-300-3p	−1.48	2.70 × 10^−2^
mmu-miR-451a	−1.39	3.62 × 10^−2^
mmu-miR-92b-3p	1.58	3.10 × 10^−2^
mmu-let-7g-5p	1.71	1.01 × 10^−2^
mmu-let-7i-5p	1.82	1.43 × 10^−2^
mmu-miR-224-5p	2.97	1.74 × 10^−2^
mmu-miR-28a-5p	4.34	3.66 × 10^−7^
**C. E12.5 vs. E13.5**		
miRNA	logFC	FDR-adjusted *p*-value
mmu-miR-300-5p	−5.39	1.74 × 10^−2^
mmu-miR-302d-3p	−3.88	1.30 × 10^−3^
mmu-miR-302a-3p	−3.71	3.03 × 10^−3^
mmu-miR-302b-3p	−3.71	3.03 × 10^−3^
mmu-miR-3063-3p	−3.56	1.30 × 10^−3^
mmu-miR-341-3p	−2.37	6.18 × 10^−4^
mmu-miR-20b-5p	−1.99	4.59 × 10^−2^
mmu-miR-335-3p	−1.73	4.22 × 10^−3^
mmu-miR-300-3p	−1.48	2.70 × 10^−2^
mmu-miR-451a	−1.39	3.62 × 10^−2^
mmu-miR-92b-3p	1.58	3.10 × 10^−2^
mmu-let-7g-5p	1.71	1.01 × 10^−2^
mmu-let-7i-5p	1.82	1.43 × 10^−2^
mmu-miR-224-5p	2.97	1.74 × 10^−2^
mmu-miR-28a-5p	4.34	3.66 × 10^−7^

Light blue and light orange background color in the table show downregulated and upregulated, respectively.

**Table 2:** Target genes of each miRNA predicted through bioinformatic analyses.

	Anti-Correlated and Predicted Target Genes
miR-28a-5p E10.5 vs. E11.5	*Ahr*, *Astn1*, *Bach1*, *Cgnl1*, *Crot*, *Cybrd1*, *Dab2*, *Entpd5*, *Frmd4a*, *Fut10*, *Gpm6a*, *Gpr155*, *Kctd12b*, *Mapk10*, *Nsg1*, *Rab18*, *Rabif*, *Rap1b*, *Sesn1*, *Slc18b1*, *Slitrk1*, *St8sia2*, *Tmem260*, *Wdr82*
miR-28a-5p E11.5 vs. E12.5	*Cd320*, *Fam169a*, *Fam43a*, *Haus2*, *Hvcn1*, *Mapk6*, *Pbk*, *Pigm*, *Pknox2*, *Rps6ka6*, *Tfam*, *Tfrc*, *Tyms*, *Zik1*
miR-302a/b/d-3p E10.5 vs. E11.5 vs. E12.5 *miR-302d-3p only	*Abca1*, Adamts5*, *Atxn1*, *Bcl11b*, *Btg1*, *Crebrf*, *Cyp26b1*, *Ednrb*, *Fam13c*, *Hivep2*, *Kif26b*, *Mapk10*, *Med12l*, *Myo1d*, *Nfia*, *Nfib*, *Npas3*, *Parp8*, *Pcdhb16*, *Pcdhb17*, *Psd3*, *Rbl2*, *Rtn1*, *Setbp1*, *Slc5a7*, *Stmn2*, *Tbc1d8b*, *Tgfbr2*, *Trim66*, *Unc5d*, *Zfp827*

## Data Availability

The original contributions presented in this study are included in the article and Supplemental Materials. Further inquiries can be directed to the corresponding author.

## Supplementary Materials

The following supporting information can be downloaded at: https://www.mdpi.com/article/10.3390/cells14141068/s1, Figure S1: Effects of candidate miRNAs on cell proliferation in O9-1 cells; Figure S2: Gene expression in O9-1 cells treated with candidate miRNA mimics; Figure S3: Gene expression in O9-1 cells treated with candidate miRNA inhibitors; Figure S4: *at*RA suppresses cell proliferation through upregulation of miR-302 family in a dose-dependent manner; Table S1: Primers used in this study.

## Abbreviations

The following abbreviations are used in this manuscript:

*at*RA	*all-trans* retinoic acid
miRNA	microRNA
MEFM	Mouse embryonic frontonasal mesenchymal
CNC	Cranial neural crest
CL/P	Cleft lip with/without cleft palate
ES	Embryonic stem
SHH	Sonic hedgehog
