# Supplementary material for: miR-302a/b/d-3p Differentially Expressed During Frontonasal Development Is Sensitive to Retinoic Acid Exposure
Source: Cells. 2025 Jul 11;14(14):1068. doi: 10.3390/cells14141068 (PMC12293701; doi:10.3390/cells14141068)
Supplement: Supplementary file 1 [file cells-14-01068-s001.zip › cells-3670233-supplementary.pdf]

Table S1. Primers used in this study

Gene	Forward Primer	Reverse Primer
Abca1	TGTTACGGCAGATCAAGCATCC	TGCTGCAGGAATCCAGAGAATG
Adamts5	GCACTACGATGCAGCCATC	CATATGGTCCCAACGTCTGCC
Ahr	CTTAGGCTCAGCGTCAGCTAC	GTGCTCTACACTGGTCCTGG
Astn1	GGAAGAGCTGAGGATCCTGC	AGTGTACAGGATCATCACCAGG
Atxn1	GAATCGAGGAGAGCCACAGC	CAGGATGACCAGCCCTGTC
Bach1	CGCTGTCGCAAGAGGAAACTTG	AGCGTTGACAGAATGTGGTCTC
Bcl11b	ATGTCCCGCCGCAAACAG	AGGCTGCTAGGCTCCTCTATC
Btg1	GCTGCAGACTTTCAGCCAGAG	CGGTAACCTGATCCCTTGCAC
Cd320	GGACCAGGACTGCTCTGATG	GAGCAACCACTGATGTTGTCAC
Cgnl1	CTTTGAAGTCCAGAGCAGCAGG	CAGTTGCTGCTGTAGTTCAGAC
Crebrf	TGATGATGAGGACCATGATGAAG	CCCTCTCTTCCGAGAAGATATGG
Crot	CACATTGCCGTGCTGTGTC	GTTCGTTCGAGCACTTCTTGTGG
Cybrd1	GAGTTTAACTGGCACCCGGTG	AAGAGCTTGCTGCACTTCCAG
Cyp26b1	AATTCCATTGGCGACATCCACC	GGTAGCTCTCAAGTGCCTCATG
Dab2	CCGCAGTCGAACTTTCTGGATC	CTGCTTGGGGGGGTGTTAC
Entpd5	GAAAGCAACGGCCGGACTC	CTAACGCTGCCATCTGGGAC
Fam13c	CTTCAGGTGCCAGGAAACGG	CCTGCTGAGTTCCAAAAGCATCC
Fam169a	GGAGAGGCCGTTGGGTTTTATTC	CCAGCATATGAAGCCCCAAATC
Fam43a	ATGAGCTGAAGCACAAGGCG	AGAGCGTTGGCTGATGTCTG
Frmd4a	GGGCCGTCGATGTCAAGTAC	GTTGAAATGAGAGGCCACGAGG
Fut10	CCGAGGCAAAGGTAAGCTGTC	CTGACTGGAGAGACTGGCAG
Gpm6a	TGCAGACCTACTTTGAGTTGGC	GCCGCAATGCCATAGATCAC
Gpr155	CCTTCTCTGGATCAGCCCTG	CCACCATTTCTCTGCACAGGAG
Haus2	AGGATACAGCAGATCTTATTCATCC	CCAATGGCAAGTTCTCCTGG
Hivep2	GGGAAAGGAGAGATTCACGGAG	TCCACGTCCTCTGACATACAC
Hvcn1	GCCGACTGAGGAAACTCTTCAG	CGTCCGGCTCGATGATCTTC
Kctd12b	GAACTCAATGTAGGTGGCCAGG	GTCCTGGATCAGTGAGCAAGG
Kif26b	CAACGTCATCCTGGCTTTGGTC	ATCATGGTGGTGCGACAGTTC
Mapk10	CGCTACCAGAACCTGAAGCC	GGGTCTGCTGAGCTTCTTAATGG
Mapk6	GACATGACTGAGCCACACAG	GAAAGGGCTTCCTCAGCTGTC
Med12l	CGACACCTGCAGTATGCAAC	TAGAGAAGGATTGTGCGCTGG
Myo1d	CTCCATGCCCGAGTTCATGG	CCCTCCCATAGATGTTCAGCAC
Nfia	GGGCTTCCAGGACAGCTTTG	GCTCCTGGACTCATGCTGTAG
Nfib	CCCAGCACTTTGTGTTCAGCC	GGATCACTGTGGCTTGGACTTC
Npas3	AGCGCACTGGCCATTGAAG	GTGCAAATACGAAGCCATCCAGG
Nsg1	GAGGAAGGCCAGGGCAAAG	GCACGTCCTCCCAAGATAGC
Parp8	GTGTGTGATGAACCACACGTG	GTCGGCAGCTTCATTCATCAC
Pbk	GGAGTCTCTCTGCCATTGGATG	TCTGCCTTGTCAGTAATGATGCC
Pcdhb16	GTAAAAGACCTGGGTCTTGGGC	GCCCACACAGTTTTTCCCTGTC
Pcdhb17	GGAAGTACCCAGAGCTGGTG	ACTTCAATGAGAACCTGTGCCG
Pigm	TTCATCAGCTGCGATCTCCTC	CCAGAAGACGCAGTAGCCAC
Pknox2	CCAGCTGGAGGCTGACAAG	TGGGTGGCCTGTTCACATTTC
Psd3	TGACCCGAATGCTGCTGTATAC	GGTTTTCCATCCTCGCTTTCCTC
Rab18	TTTGCACGCAAGCATTCTATGT	TTGTTCTGGTTCTCACTTTCCC
Rabif	CAGCCAGGGACTGCTCTTTTC	CCTGGAGGAGATCACCATCAG
Rap1b	CCACTGTCTTGCTAGGTTCTGAC	CTAAGAGAGCAGATTCTGCGGG
Rbl2	GCCTCAGGAATGCACCCAG	TAGCCTGAGTTGGATCTCGGG
Rps6ka6	GATTTCCTCAGGGGAGGAGATG	TGTAGATGATCCAAAGCCAGGG
Rtn1	GTCGCCATGGAAACTGCATC	AGGTGGATAGCAGACGTCAG
Sesn1	CACACCTTCAGACCGCCTTC	GAGAGCTTCCACCTCAAAGAAGG
Setbp1	GCCCACCTGAGATCAAGATCAC	GCTTTGAGGTCGCTGGCTG
Slc18b1	GCATAGTCCTGCTGATGATACC	AGATGATCACGAAGGCTAGGAG
Slc5a7	TTCATCCCTGCACTGATGGG	GGCAATGAGTGCAGAGACAATG
Slitrk1	GGCTTGCATGAGATCGTTCC	GCCTGGAGGTATTCCAGATCG
St8sia2	ATGGAGACACAACCAGACGC	GTCTCCAGGCTTCAGGGTC
Stmn2	AGCTCCACGAACTCTAGCTTC	TGCCAACTGCTTCAGCACC
Tbc1d8b	TCGAGGCTACGGGGAAGAAG	ATCTGGTGTCTGGTGTAGGATG
Tfam	GCTTGGAAAACCAAAAAGACCTCG	GCTTTTCCTCAGGAGACAGATTTTTC
Tfrc	GTCCAGTGTGGGAACAGGTC	CCAAAGTCGCCTGCAGTCC
Tgfbr2	AAAGAAAAGGGCGGGCGAGAC	GACCAACAACAGGTCGGGAC
Tmem260	GATTCGGAACCTGGAGTGGC	CTGAGGCATGGAGTCCAGAAG
Trim66	CCAGACATCTGGGGAGGAAAC	AGCTGTCAGGCTGCTCAC
Tyms	GGCTTCAAGAAGGAGGACCG	CCCTTCCAGAACACTCGTTTGG
Unc5d	CAAGGCAGGGAGGTTCCTATTG	CATCCTGTTCGGAGTCGATGG
Wdr82	TTAGTCCCAATGGCGAGACG	GGGTGTATCTGATGAGGTCCAC
Zfp827	CCAGGAGGAAGCAGGAGC	CTTCCCCATAGGACGCTTCTG
Zik1	GAGACTCAGAGGCTCCTGTAC	CGCTCGTGTAGAAACCCTTCG
Gapdh	AACTTTGGCATTGTGGAAGG	ACACATTGGGGGTAGGAACA

Figure S1

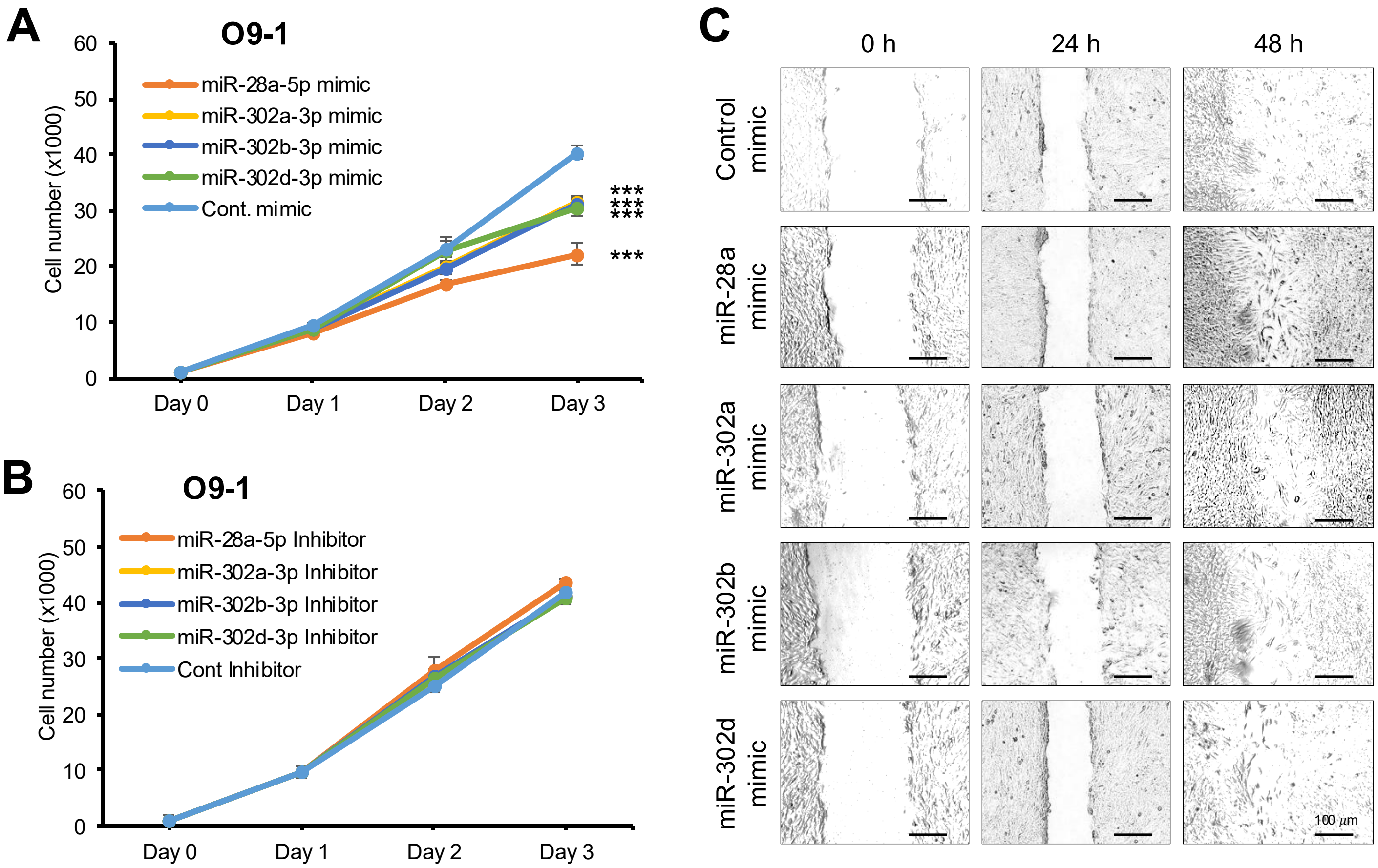

Figure S1. Effects of candidate miRNAs on cell proliferation in O9-1 cells. (**A, B**) Cell proliferation assays in O9-1 cells treated with the indicated miRNA mimic (A) or inhibitor (B) for three days. ***p < 0.001. (**C**) Cell migration assays at 0, 24, and 48 hours in O9-1 cells treated with the indicated miRNA mimic. Scale bars, 100 μm.

Figure S2

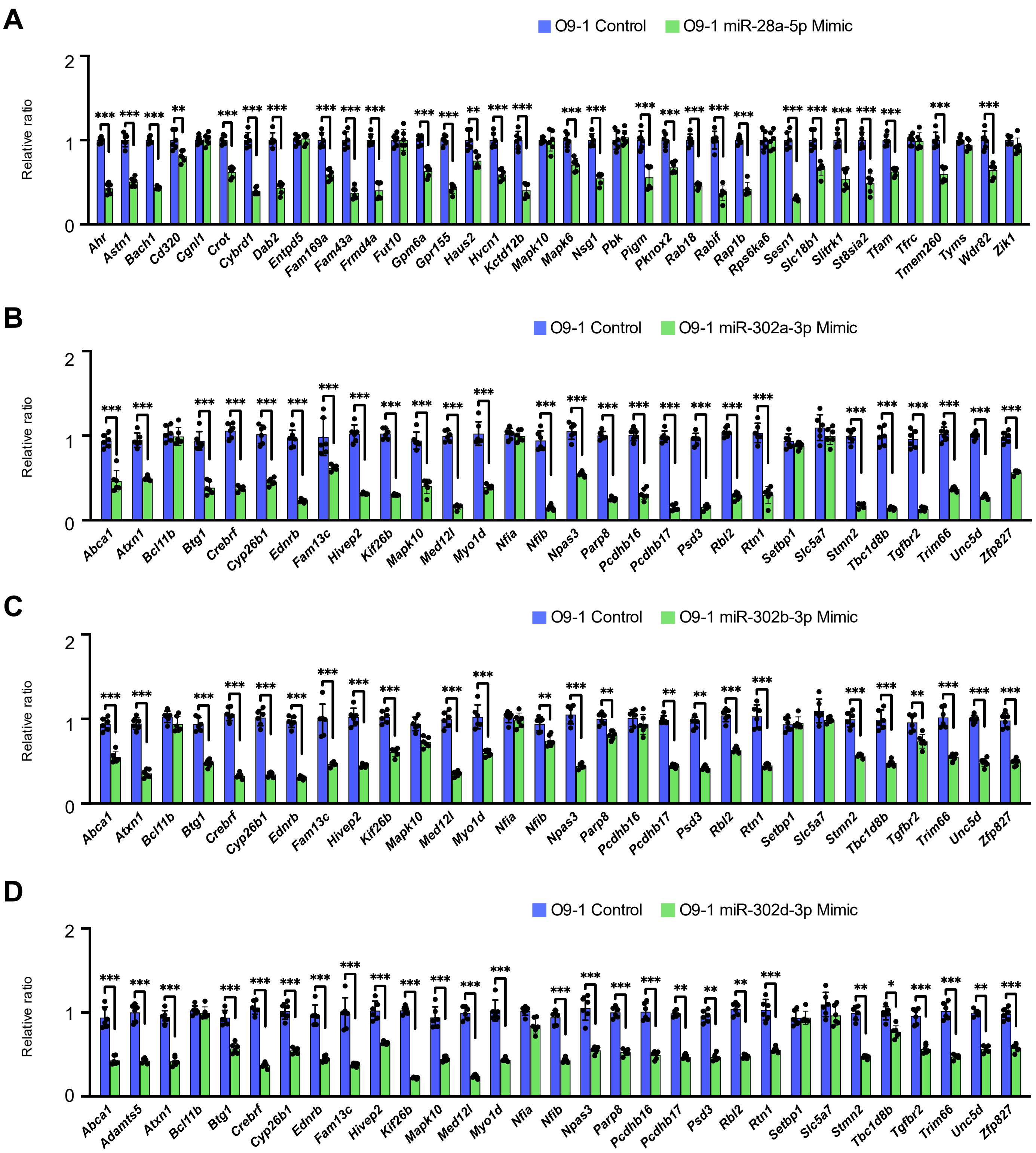

Figure S2. Gene expression in O9-1 cells treated with candidate miRNA mimics. (**A-D**) Quantitative RT-PCR for target genes in MEFM cells treated with miR-28a-5p mimic (A), miR-302a-3p mimic (B), miR-302b-3p mimic (C), and miR-302d-3p mimic (D) for 24 hours. **$p < 0.01$, ***$p < 0.001$.

Figure S3

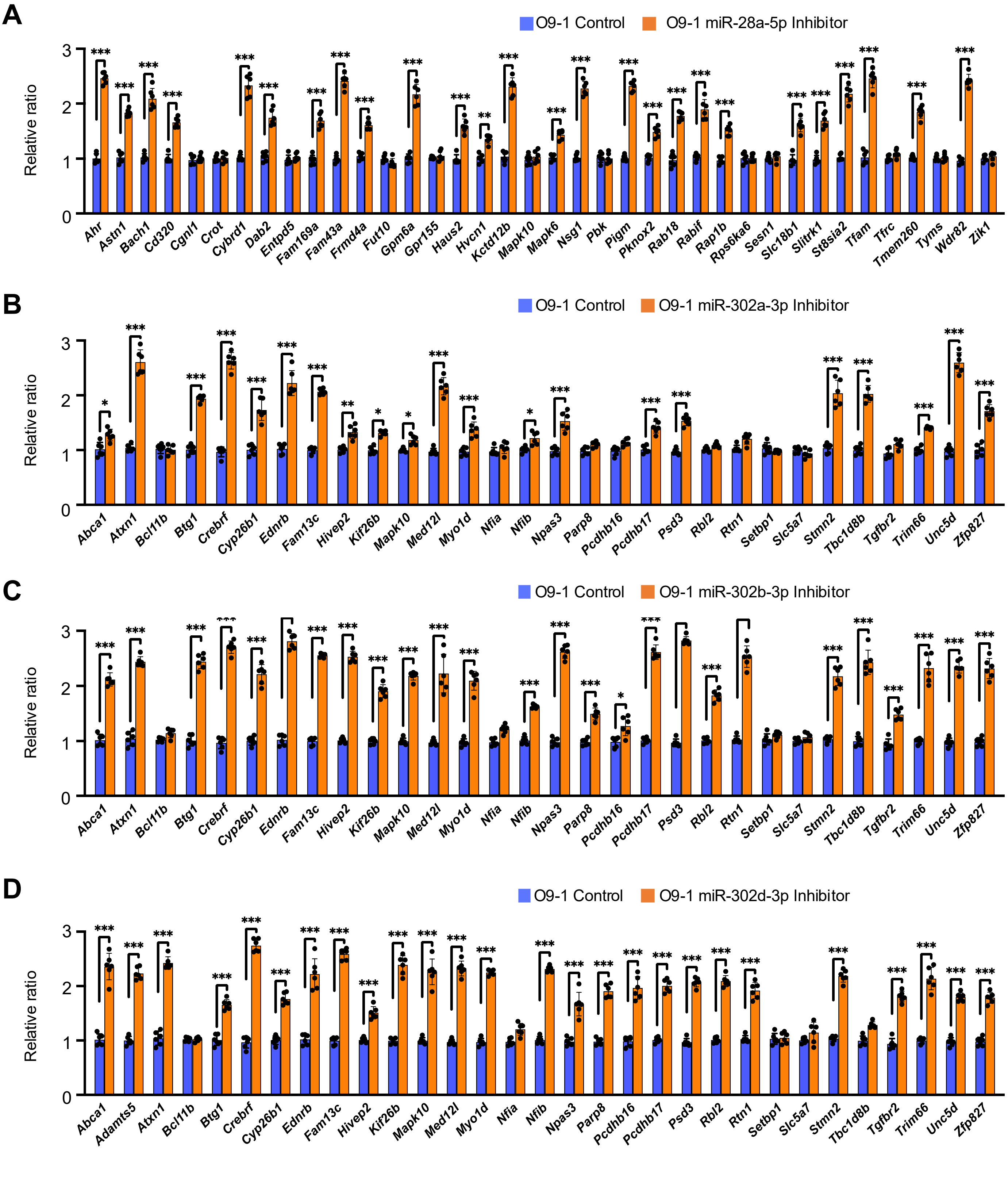

Figure S3. Gene expression in O9-1 cells treated with candidate miRNA inhibitors. (**A-D**) Quantitative RT-PCR for target genes in O9-1 cells treated with inhibitors for miR-28a-5p (A), miR-302a-3p (B), miR-302b-3p (C), and miR-302d-3p (D) for 24 hours. *$p < 0.05$, **$p < 0.01$, ***$p < 0.001$.

Figure S4

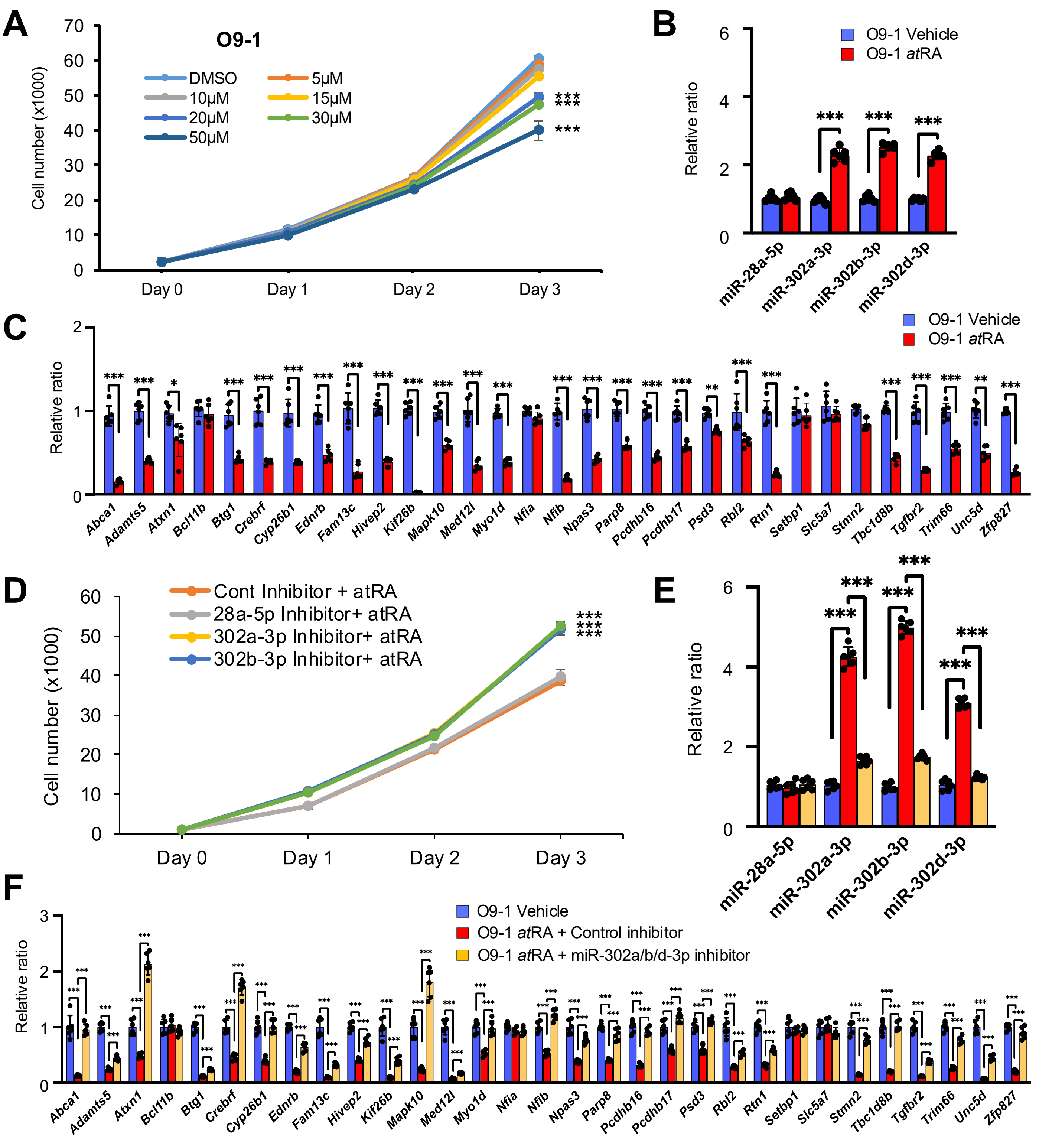

Figure S4. *at*RA suppresses cell proliferation through upregulation of miR-302 family in a dose-dependent manner. (**A**) Cell proliferation assays in O9-1 cells treated with *at*RA at 5 to 50 μM for three days. (**B**) Expression of miR-302 family and miR-28-5p in O9-1 cells treated with 50 μM *at*RA. (**C**) Quantitative RT-PCR for target genes in O9-1 cells treated with 50 μM atRA. ****p* < 0.001. Each treatment group was compared to a vehicle control group. (**D**) Cell proliferation assay in O9-1 cells treated with miRNA inhibitors for miR-28a-5p or miR-302a/b/d-3p under 50 μM *at*RA for three days. (**E**) miRNA expression in O9-1 cells treated with each miRNA inhibitor under 50 μM *at*RA. (**F**) Quantitative RT-PCR for target genes in O9-1 cells treated with miR-302a/b/d-3p inhibitors under 50 μM *at*RA. ****p* < 0.001.
